# A New Method of Treating General Suppurative Peritonitis

**Published:** 1897-09

**Authors:** 


### A New Method of Treating General Suppurative Peritonitis.

Finney (Johns-Hopkins Hospital *Bulletin*, July) reports five cases of general suppurative peritonitis (including one of typhoid perforation) successfully treated by a new method. The same procedure was followed in a sixth case, and although the patient was at the same time *in extremis*, life was prolonged for thirty-six hours. It is not claimed that the principle involved in the operation is a new one, but only that the manner of its application is original. The operation consists, first, in a sufficiently long incision to admit of easy access to all parts of the peritoneal cavity. Next the coils of small intestine are quickly withdrawn, beginning with the worst coils. All or as much of the small intestines as is necessary is removed and placed outside of the abdomen and covered with warm gauze or towels. Then the peritoneal cavity is thoroughly and systematically wiped out with large pledgets of gauze wrung out of hot salt solution, particular attention being paid to the pelvic portion. In some cases it may be well, in addition, to flush out the cavity with warm salt solution, but this is rarely necessary. Next the small intestine should be systematically examined loop by loop while still outside the abdomen and rendered macroscopically clean by wiping with gauze compresses wrung out of hot salt solution. It is necessary to wipe with considerable force at times in order to remove adherent flakes of partly organized lymph. This should be done thoroughly and conscientiously, as upon it depends, in a large measure the success of the operation. The cleansing process is facilitated, and the shock of the operation is lessened if the wiping of the intestinal coils is carried on under a constant irrigation of warm salt solution. After being cleansed macroscopically of all foreign material, pus, feces, lymph, etc., the intestines should be replaced in the abdomen, the worst or sutured coil being the last or most superficial, in order that it may be the better drained by being packed about with gauze if necessary. The abdominal wound is then tightly closed, just enough room being left between two sutures for the gauze drain. If there are any evidences of distension or pain, the Paquelin cautery should be thoroughly applied to the abdomen and the bowels moved early by calomel in broken doses, followed by salts and a turpentine enema. An experimental study by Elting and Calvert (Johns-Hopkins Hospital *Bulletin*, July) of perforative peritonitis, submitted to the foregoing treatment, showed that mere mechanical irritation of the peritoneal surfaces will lead to the formation of adhesions; that peritonitis in dogs, caused by a perforation of the intestine, is of an intensely hemorrhagic character, and if left to itself, rapidly proves fatal; that generalized peritonitis of this character in dogs can be cured as late as six and a half hours after the operation by the cleansing method described, and that mere closure of the perforation, without this cleansing operation will rarely, if ever, cure a case of generalized peritonitis in dogs.—*Jour. Am. Med. Asso.*

